# Esterification of Cellulose with Long Fatty Acid Chain through Mechanochemical Method

**DOI:** 10.3390/polym13244397

**Published:** 2021-12-15

**Authors:** Jacqueline Lease, Tessei Kawano, Yoshito Andou

**Affiliations:** 1 Department of Biological Functions Engineering, Graduate School of Life Science and Systems Engineering, Kyushu Institute of Technology, 2-4 Hibikino, Wakamatsu-ku, Kitakyushu, Fukuoka 808-0196, Japan; lease.jacqueline708@mail.kyutech.jp (J.L.); kawano.tessei758@mail.kyutech.jp (T.K.); 2 Collaborative Research Centre for Green Materials on Environmental Technology, Kyushu Institute of Technology, 2-4 Hibikino, Wakamatsu-ku, Kitakyushu, Fukuoka 808-0196, Japan

**Keywords:** microcrystalline cellulose, mechanochemical esterification, long fatty acid chain, oleic acid, ionic liquid, magnetic mortar and pestle

## Abstract

Mechanochemical reaction, a green synthetic esterification route was utilized to prepare long-chain cellulose esters from microcrystalline cellulose. The influence of reaction conditions such as reaction temperature and time were elucidated. Only low dosage of oleic acid, 1-butyl-3-metylimidazolium acetate, and *p*-toluenesulfonyl chloride were required. The success of modification reaction was confirmed by Fourier transforms infrared spectroscopy as a new absorbance peak at 1731 cm^−1^ was observed, which indicated the formation of carbonyl group (C=O). Solid-state nuclear magnetic resonance was also performed to determine the structural property and degree of substitution (DS) of the cellulose oleate. Based on the results, increasing reaction temperature and reaction time promoted the esterification reaction and DS. DS values of cellulose oleates slightly decreased after 12 h reaction time. Besides, X-ray diffraction analysis showed the broadening of the diffraction peaks and thermal stability decreased after esterification. Hence, the findings suggested that grafting of oleic acid’s aliphatic chain onto the cellulose backbone lowered the crystallinity and thermal stability.

## 1. Introduction

Lignocellulosic biomass is garnering attention among researchers as it is a readily renewable and environmentally benign natural source. Among lignocellulosic materials, cellulose is the most abundant biopolymer in nature composed of anhydroglucose units, which is linked by β-1,4-glucosidic linkages [1]. Cellulose materials offered advantages of biodegradability, biocompatibility, high surface area, low cost, high thermal stability, and high mechanical properties [2,3].

Nevertheless, the absence of thermal transition makes cellulose non-processable and the presence of inherent strong intra- and intermolecular hydrogen bonding through hydroxyl groups often results in pronounced aggregation [4]. The agglomeration led to cellulose being insoluble in water and most of organic solvents. To overcome the aforementioned problems, esterification has been demonstrated as an effective approach to improve the aggregation and thermoplastic behavior of cellulose [5].

Conventionally, chemical esterification of cellulose was carried out via pyridine-acyl chloride or anhydride reactions. These reaction systems produced hydrochloric acid as a byproduct, which resulted in cellulose degradation and is environmentally harmful [6,7]. To date, a number of non-derivative solvent systems, such as *N*,*N*-dimethylacetamide-lithium chloride (DMAc-LiCl) [8], *N*-methylmorpholine *N*-oxide (NMMO) [9], and dimethyl sulfoxide-tetramethylammonium fluoride (DMSO-TBAF) [10] have been found to be efficient in the cellulose dissolution process. However, several drawbacks from the solvent systems include high cost, the required large amount of chemicals, volatility, toxicity, and difficulty in solvent recovery [11,12].

Among potential new solvents, the application of ionic liquids (ILs) as a solvent system has been shown to be a feasible yet effective system. The usage of ILs as suitable solvents produced no toxic or explosive gases and can be recycled for repeated usage [13]. Their negligible vapor pressure, low viscosity, improvement of selectivity and yields, excellent thermal stabilities, as well as excellent dissolution performance for cellulose have made them solvents of choice [14,15]. According to Kakuchi et. al. [16], IL such as 1-ethyl-3-methylimidazolium acetate (EmimOAc) can offer dual functionalities by acting concurrently as a facile solvent for cellulose and as an activating reagent for esterification reaction of cellulose hydroxy groups. In this work, 1-butyl-3-metylimidazolium acetate (BmimOAc) was used as both reaction media and catalyst for the esterification reaction.

Besides ILs, mechanochemical esterification is another green synthetic process used to address the setbacks of environment burden. Mechanochemistry is a chemical reaction induced and sustained by the use of mechanical force (grinding or milling) [17,18]. Ball milling is a well-known technique for benchtop pilot scale study, so it is possible to realize the value of cellulose in industrial sectors due to the efficient chemical transformation generated through mechanical forces [18,19,20]. Cellulose esterification employing mechanochemical methods has been studied previously, but most of the literature focused on short-chain fatty acid in the presence of solvent during the reaction [5]. Long-chain oleic acid (OA) is predicted to be the best substitute to serve as esterifying agent for cellulose esterification as it does not produce harmful by-products. In addition, oleic acid has excellent flexibility, wider thermal process window, low melting point, easy processing, renewability, and hydrophobicity [5,21]. Herein, mechanochemical esterification with long chain OA in IL is proposed as a facile yet environmentally friendly protocol to synthesize cellulose oleate (CO).

The novel aspect of this work details the synthesis of CO in a green, simple, and scalable way by controlling the reaction conditions. Furthermore, degree of substitution (DS), thermal stability, and the chemical structure of the CO were identified.

## 2. Materials and Methods

### 2.1. Materials

Microcrystalline cellulose (MCC) powder with a particle size of 20µm was obtained from Sigma-Aldrich, St. Loius, MO, USA and was dried under vacuum conditions at 60 °C for 6 h before use. A total of ≥95% of 1-butyl-3-metylimidazolium acetate (BmimOAc) was also purchased from Sigma-Aldrich. Oleic acid (OA) was supplied by Wako Pure Chemical Industry, Osaka, Japan. *p*-Toluenesulfonyl chloride (TsCl), which acts as an activating agent, was provided by Tokyo Chemical Industry (Tokyo, Japan) and stored in a desiccator cabinet with silica gels to prevent water absorption. All the reagents were used as received without further purification or treatment.

### 2.2. Preparation of Cellulose Oleate (CO)

**Scheme 1:**
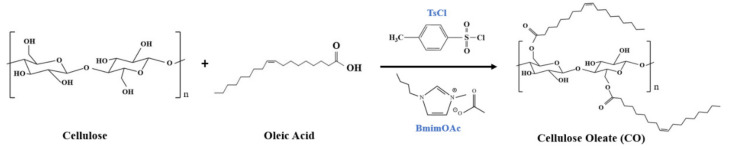
Schematic representation of the mechanochemical esterification reaction of cellulose in BmimOAc with OA, and TsCl as the activating reagent.

Modification of cellulose with OA in TsCl/BmimOAc system via mechanochemical esterification was depicted (Scheme 1). Before functionalization, MCC powder was dried for 8 h at 70 °C before use to remove any trapped moisture from the atmosphere. A total of 0.5 g of MCC (3 mmol), 5 g of OA (18 mmol), and 3.6 g of BmimOAc (18 mmol) were added and kneaded in a magnet mortar with a constant rotational speed of 150 rpm (100 VAC, 15 W). After 1 h, 3.5 g of TsCl (18 mmol) was added into the mixture to induce the hydroxyl groups of the cellulose backbone into better leaving groups. The mixture was continuously stirred at different reaction temperatures (50, 80, and 100 °C) and reaction time (4, 12, and 24 h). After the mechanochemical esterification process, the product was precipitated in acetone, followed by Soxhlet extraction with methanol to remove unreacted substances for 8 h. The CO was filtered and washed with ethanol twice to completely remove any unreacted oleic acid. Lastly, the modified cellulose was vacuum-dried at 80 °C and stored at ambient conditions.

### 2.3. Characterizations

#### 2.3.1. Fourier Transform Infrared (FTIR) Spectroscopy

The chemical structures of as-prepared CO were analyzed by FTIR spectroscopy (Nicolet iS5, Thermo Fisher, Waltham, MA, USA). A total of 2 mg of dried sample was mixed thoroughly with 100 mg potassium bromide (KBr) in a mortar. The mixture was then pressed into a pellet until a translucent film was obtained. Sixteen scans were taken for each run in a wavenumber range of 400–4000 cm^−1^.

#### 2.3.2. Solid-State Nuclear Magnetic Resonance (ssNMR)

Solid-state 13C NMR spectra of CO was recorded by using ssNMR (JNM-ECA-500 MHz II, JEOL Resonance, Tokyo, Japan) spectrometer. Samples were placed in a zirconia rotor and spun at 6 kHz with 2000 scans. The temperature was set to 298 K and the degree of substitution of cellulose oleates (DS_NMR_) were calculated from a ratio of the integrals of NMR peaks using the following formula [22]:

DS_NMR_ = (n_cel_ × I_oleoyl_) / (n_oleoyl_ × I_cel_), (1)

where, I_oleoyl_ and I_cel_ are the integrations of peaks corresponding to oleoyl carbons and cellulose carbons, respectively. n_cel_ and noleoyl are the number of carbon atoms in cellulose (n_cel_ = 6) and in the corresponding acetyl group (n_oleoyl_ = 18), respectively.

#### 2.3.3. Thermogravimetric Analysis (TGA)

The thermal stability of the unmodified cellulose and modified cellulose were determined using EXSTAR TG/DTA 6200 (SII Nanotechnology Inc., Chiba, Japan) under constant nitrogen flow (100 mL/min). The samples (5–10 mg) were heated from 30–500 °C with a heating rate of 10 °C/min.

#### 2.3.4. Scanning Electron Microscopy (SEM)

Morphologies of the cellulose before and after functionalization were performed using a JCM-6000 SEM (JEOL, Tokyo, Japan). Before the observation, the samples were mounted on an aluminum stub and coated with carbon first using vacuum sputter-coater to improve conductivity and prevent charging of the samples. SEM images were obtained at an acceleration voltage of 15 kV.

#### 2.3.5. X-Ray Diffraction (XRD) Analysis

XRD measurements were characterized on a Rigaku Miniflex II diffractometer using CuKα radiation (λ = 0.154 nm). The samples were exposed to the X-ray beam with the X-ray generator running at 30 kV and 15 mA. Scattered radiation was detected at ambient temperature in the angular region (2θ) of 3° to 70° at a rate of 10°/min and a step size of 0.02. The crystallinity index (CrI) of the cellulose derivatives was calculated by the following equation [23]:

CrI (%) = [(I_002_ − I_am_)/I_002_ × 100%], (2)

where, I_002_ is the maximum intensity of the (002) crystal plane reflection of the cellulose and Iam is the minimum intensity between the (002) and (101) peaks.

Crystallite size was calculated by using Scherrer’s formula [23]:

Crystallite size = 0.9 λ/(β cos θ),(3)

where, λ is the wavelength of X-ray beam with 0.154 nm, β is the full width at half maximum (FWHM), and θ is the angle of beam reflection.

#### 2.3.6. Differential Scanning Calorimetry (DSC) Analysis

DSC was carried out with an EXSTAR DSC 6220 (SII Nanotechnology Inc., Tokyo, Japan). The sample was first heated from 30 to 150 °C at a scanning rate of 10 °C min^−1^ to provide the same thermal history before measurements. The temperature was maintained at 150 °C for 1 min and then quenched to −50 °C. The second heating scan was conducted from −50 to 200 °C at a scanning rate of 10 °C min^−1^ to examine the glass-transition temperature (Tg). The results of Tg from the Figure S3 were obtained from second heating.

## 3. Results and Discussion

### 3.1. Preparation of Cellulose Oleate (CO)

**Figure 1:**
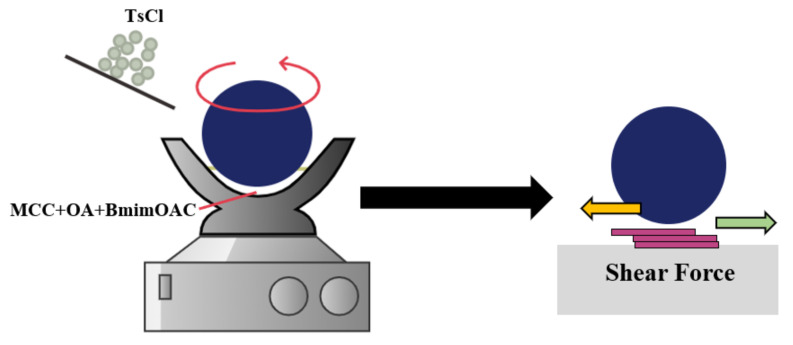
Mechanochemical esterification of MCC by using magnetic agate mortar and pestle.

In this work, esterification of microcrystalline cellulose (MCC) with oleic acid (OA) in *p*-Toluenesulfonyl chloride (TsCl) and 1-butyl-3-metylimidazolium acetate (BmimOAc) system was realized by mechanochemical method (Figure 1). When MCC was subjected to the intense milling, the stable crystalline structure and strong inter- and intramolecular hydrogen bonding can be destroyed and the steric effect of OA (long fatty acid chain) was also weakened. This scenario might lead to the increase of the reactivity of hydroxyl groups in the MCC.

Here, green mechanochemical reaction could be carried out with low amount of catalysts and solvents due to the physical shearing force if compared to conventional chemical esterification. The binding sites in cellulose induced by intense milling can rapidly react with oleate group, assisting by combining the milling and esterification reaction in the mortar. The chemical structures, thermal stabilities, and degree of substitution of MCC with different reaction temperature and time were analyzed and investigated.

### 3.2. Chemical Structure of Cellulose Oleates (CO)

**Figure 2:**
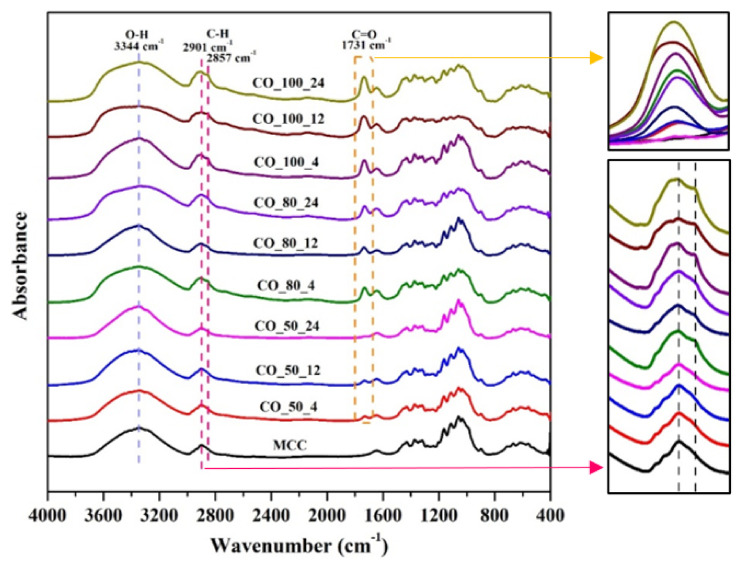
FTIR spectra of cellulose oleates synthesized at different reaction time (4, 12, and 24 h) and reaction temperature (50, 80, and 100 °C).

The chemical structures of COs were characterized by FTIR and the results were presented in Figure 2. FTIR spectra of the COs showed clear evidence that esterification had occurred from the considerable differences with the unmodified cellulose. After mechanochemical esterification, a new absorbance peak at 1731 cm^−1^ was recorded, which was assigned to the stretching vibration of the carbonyl group (C=O) [24]. From the spectra, the carbonyl peaks increased linearly over reaction temperature.

Besides, it can be clearly observed that the single absorptive band in the unmodified cellulose at 2901 cm^−1^ was changed to double absorptive bands (2901 cm^−1^ and 2857 cm^−1^) in the modified cellulose, which could be attributed to the introduction of more methylene groups (C–H bonds) from the OA [25]. The characteristic peak of the unsaturated group (H–C=C) of the oleic acid was also observed at 3009 cm^−1^.

The decrease in intensity of the broad band at about 3344 cm^−1^, assigned to the cellulose O–H vibration was additional proof of the successful esterification. The decreasing peak intensity in the cellulose hydroxyl region suggested the substitution of hydroxyl group with fatty acid, especially in high reaction temperatures, 80 and 100 °C. The peak changes of O–H vibration were not obvious for the modified cellulose at lower temperature (50 °C), which can be explained by the low rate of fatty acid side chain grafted on the cellulose.

### 3.3. The Effect of Reaction Conditions on DS of Cellulose Oleate (CO)

Nuclear magnetic resonance (NMR) spectroscopy is divided into liquid-state NMR (lsNMR) and solid-state NMR (ssNMR). After functionalization, CO still cannot be dissolved in organic solvents, but well-dispersed in dimethylformamide (DMF) and tetrahydrofuran (THF) (Figure S1 and Table S1). In the Figure S2, CO also showed better dispersity compared to MCC in DMF and THF after standing for one day. This scenario indicated that the hydroxyl group of cellulose backbone was partially removed and became less polar. In order to obtain reliable results of molecular structures, 13C ssNMR spectroscopy was used for further investigation.

**Figure 3:**
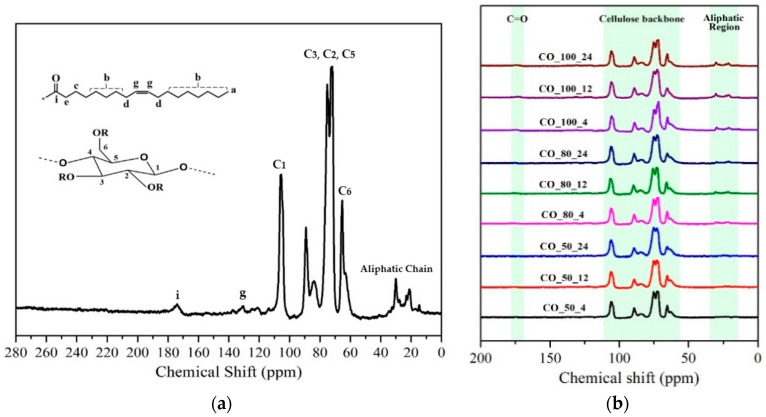
Solid state NMR (ssNMR) spectra of cellulose oleates (**a**) 100 °C and 24 h; (**b**) various reaction temperature and time.

Figure 3a shows the NMR spectra of 100 °C, 24 h cellulose sample. The signals at 105.65, 89.19, 75.24, 72.74, and 65.24 ppm were assigned to the carbon atoms on the anhydroglucose (AGU) unit at C1, C2, C3, C4, C5, and C6, respectively. The appearance of signal at 174.18 ppm was carboxylic carbon (C=O) and the signal at 130 ppm was observed as vinyl group of carbon (C=C). Besides, methyl (–CH_3_) and ethyl groups (–CH_2_) at 14 to 35 ppm supported the notion of esterification reaction [26].

The substitution level of fatty acid is the average number of oleoyl groups per anhydroglucose (AGU) unit, ranging from zero to three. Based on the peak assignments and the corresponding integrals, the DS of the oleoyl group on AGU units were summarized in Table 1. The ratio between the number of carbon atoms in the AGU unit C-2,3,4,5,6/C-1 was fixed at 5:1 and DS were calculated based on the integral of C-1 to oleoyl group ratio [22]. The area under the graph of NMR peaks in relation with standard allows the calculation of DS precisely.

The NMR spectra of modified cellulose under different reaction conditions with their respective signal assignation is presented in Figure 3b. The calculated DS values ranged from 0.001 to 0.210. The findings showed that the modification via mechanochemical method is limited to the surface or the outer layer of the cellulose bundles. Low DS indicated that only partial intramolecular interaction occurred and the ultrastructure of cellulose oleates might not be affected by the reaction [27].

Based on the DS values of COs, increasing the reaction temperature and reaction time were favorable to improve the DS [6,28]. However, the DS slightly decreased after 12 h. This trend could be explained by the possible competition between the esterification reaction and the partial hydrolysis of the ester groups formed as the byproduct of the reaction is water. The presence of moisture in the reaction medium is detrimental to the process [27].

In the case of CO_100_12 and CO_100_24, the DS values of both samples did not show significant changes, which are 0.210 and 0.204. The findings suggested that the hydrolysis process can be prevented at an elevated temperature (100 °C) and the saturation point is reached at 12 h.

### 3.4. Crystal Structure of Cellulose Oleates (CO)

**Figure 4:**
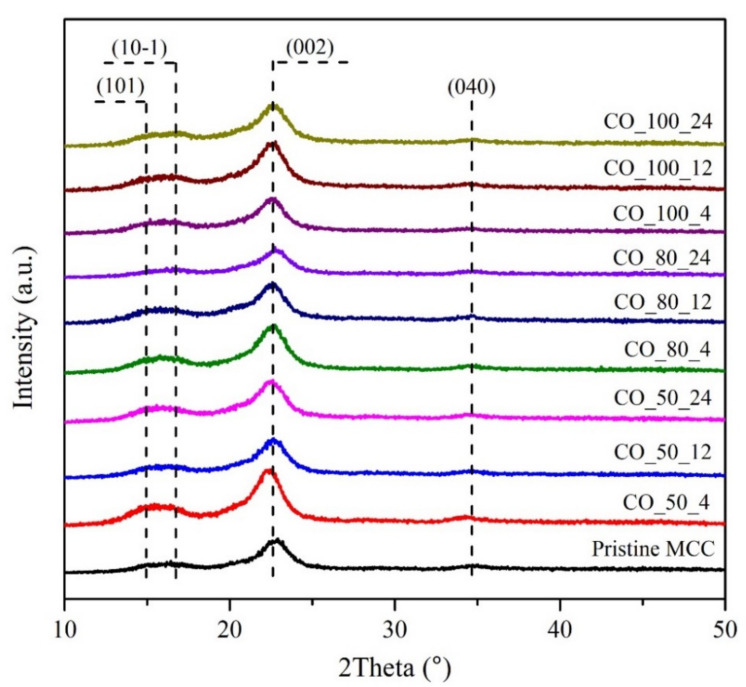
X-ray diffractograms patterns of MCC and modified cellulose (COs).

To gain further insights into the structure changes caused by mechanochemical treatment, X-ray diffraction analysis (XRD) was conducted and the respective patterns of all cellulose samples were shown in Figure 4.

The pristine cellulose and mechanochemical-treated cellulose showed the main reflection peaks at around 2θ = 14.8°, 16.4°, 22.7°, and 34.7°, which are normally ascribed to the (101), (101ˉ), (002), and (040) diffraction planes, respectively. Based on the diffraction peak angles, they were exhibited characteristic peaks as cellulose I. The XRD patterns of modified cellulose remained unchanged in comparison to the microcrystalline cellulose, indicating that the reaction only partially took place without affecting the inner structure of cellulose [29]. The modification is posited to occur at the amorphous regions of the cellulose.

The crystallinity index (CrI), full width at half maximum (FWHM), and crystallite size of each sample were calculated according to their corresponding XRD diffractogram. The results are presented in Table 2.

From Table 2, it was observed that the crystallinity index of pristine MCC is higher than COs based on the Segal calculation. The crystallinity index of COs slightly decreased after esterification due to the attachment of oleoyl side chain [30]. Oleic acid moieties have long alkyl chains, which severed the intra- and intermolecular hydrogen bonds of the cellulose result in a less crystalline structure [31].

The trend of crystallite size of modified MCCs obtained in this study did not support findings from previous studies [23,31]. It was observed previously that the crystallite size and crystallinity is inversely proportional to the reaction time and temperature. The FWHM of the modified cellulose obtained via the mechanochemical method is expected to decrease with the prolong reaction time and temperature. Nonetheless, findings in this study did not abide the observed trends as the crystalline size and crystallinity did not have significant changes. The low DS of cellulose oleate is posited to be one of the reasons that the crystallite size and crystallinity remain almost unchanged, which are in the range of 4.01–4.89 nm and 84.63–78.77%. Further study needs to be carried out to investigate the unexpected behavior.

### 3.5. Thermal Stability of Cellulose Oleate (CO)

In this work, differential scanning calorimetry (DSC) and thermogravimetric analysis (TGA) were used to examine the thermal stability of CO. DSC was performed to investigate the macroscopic manifestation of the macromolecular chains motions by determining glass transition (Tg). Based on Figure S3, CO_50_12 and CO_80_12 had no obvious glass transition, which exhibit the same behavior as pristine MCC.

Theoretically, due to the strong inter- and intramolecular hydrogen bonding, the unmodified MCC has no Tg prior to its decomposition [32]. As the substitution rate of oleoyl increased, the oleoyl side chain weaken the hydrogen bonding interaction. CO_100_12 has relatively higher DS showed one major glass transitions at 116.5 °C, which was attributed to the motion of cellulose backbone and oleoyl side chain, respectively [20].

From the thermogravimetric analysis, the thermal stability of modified cellulose decreased after surface modification. Modified MCCs started to decompose at lower degradation temperature (236–294 °C) than pristine MCC (337 °C).

**Figure 5:**
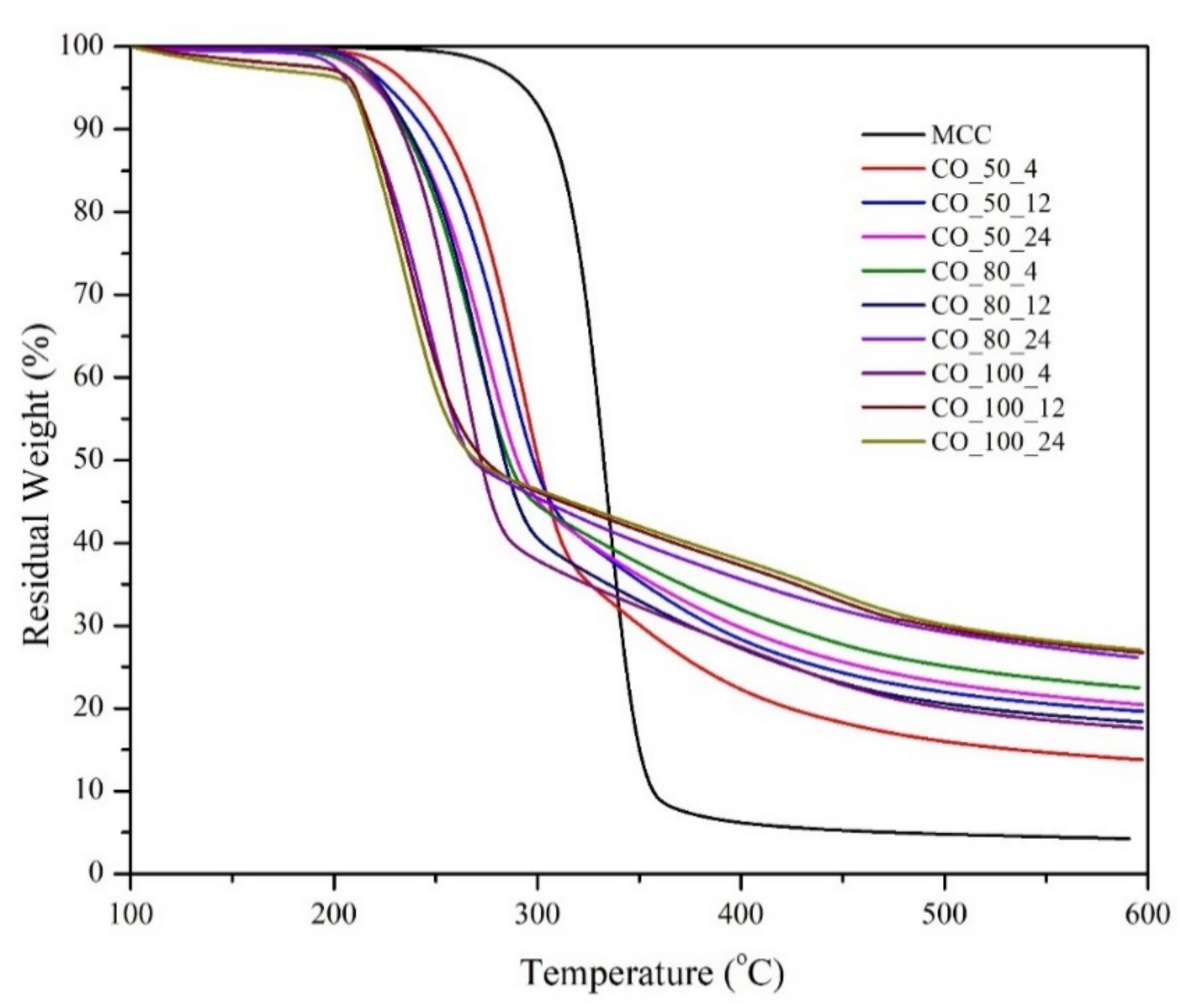
TGA thermograms of unmodified MCC and modified MCC.

Thermal stability of modified cellulose was affected by the disruption of hydrogen bonding, which decreased the crystallinity of the cellulose after the substitution with fatty acid [31]. In addition, the intensive mechanical force that promoted decrystallization is expected to be another reason. The small particle size of CO (Figure 5) enabled more exposure to heat due to their larger surface area. Therefore, thermal stability decreased after esterification took place [29].

In general, thermograms of cellulose esters showed two main degradation steps. However, all of the samples in this study showed one-step degradation curve except CO_100_12 (T_d2_ = 445 °C) and CO_100_24 (T_d2_ = 455 °C). According to Uschanov et al. [33], the absence of the second transition at higher temperature is because of the low amount of oleoyl group grafted on the cellulose. Double bonds of OA are prone to crosslinking reaction and the lack of fatty acid during heating, which inhibit the decomposition process.

For the thermal profile, the first degradation of cellulose oleate was attributed to the esterified cellulose, while the second degradation indicated the formation of new ordered region, which corresponds to the crystallization of oleoyl chains. Oleic acid contained one double bond between C9 and C10 with the cis configuration, which does not favor the formation of an ordered structure [23]. Hence, only high DS cellulose oleate exhibited two main thermal degradation steps in this study.

### 3.6. Surface Morphology of Cellulose Oleate (CO) by SEM Analysis

**Figure 6:**
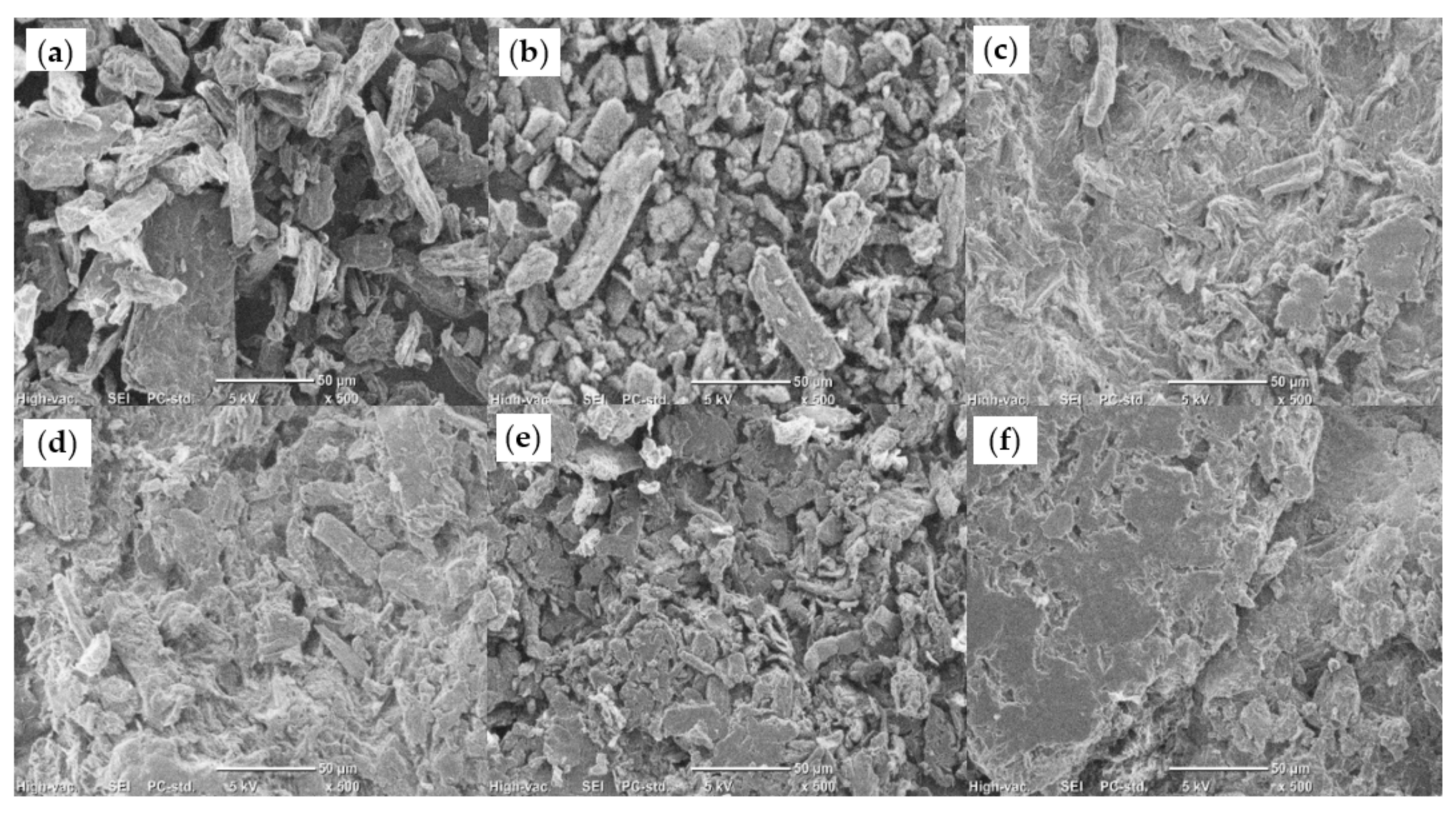
SEM images of (**a**) pristine MCC; (**b**) cellulose oleate 50 °C 12 h; (**c**) cellulose oleate 100 °C 12 h; (**d**) cellulose oleate 80 °C 4 h; (**e**) cellulose oleate 80 °C 12 h; (**f**) cellulose oleate 80 °C 24 h.

The surface morphologies of pristine cellulose and COs prepared under varying conditions can be clearly observed from their SEM micrographs (Figure 6).

The pristine microcrystalline cellulose composed of smooth rod-like cellulose bundles without small fragment on the surface [34,35,36]. The images of the modified cellulose Figure 6b–f showed that esterification increased the surface roughness of the cellulose bundles as the reaction temperature and reaction time increased. Pristine MCC was remarkably turned into smaller and irregular particles after mechanical grinding.

COs with milling temperature of 80 and 100 °C exhibited loose and porous structures [37]. Besides, their surface displayed homogenous appearance if compared to pristine MCC and COs with milling temperature of 50 °C. Interruption of the cellulose backbone can explain this observation as a result of the reaction of hydroxy group (–OH) of MCC with OA. Larger surface area could provide a facile access for oleoyl side chain to contact with the hydroxyl groups of cellulose, which significantly enhanced the mechanochemical esterification reaction [38].

## 4. Conclusions

In this research, cellulose oleates were successfully prepared via a green approach of mechanochemical esterification with a small amount of oleic acid and ionic liquid. By controlling the reaction time and temperature, various DS values (0.001–0.210) of cellulose oleates were obtained and the crystalline structure of modified cellulose was not altered via functionalization. Increasing DS of cellulose oleate promoted decrystallization of cellulose, but it retained the cellulose I structure as possessed by pristine MCC. The fine tuning of the reaction conditions will be beneficial in various industries requiring cellulose with certain properties. This reported method enables the preparation of cellulose ester with different properties for their intended applications. More reaction conditions will be considered in the future, so that high DS of cellulose can be obtained easily using magnetic mortar method. This fundamental study provides a simple yet effective method for functionalizing cellulose, opening up numerous opportunities for future research to address the drawbacks of pristine cellulose.

## Figures and Tables

**Table 1:** Degree of substitution (DS) of cellulose oleates at different reaction conditions.

Samples	Temperature (°C)	Reaction Time (h)	DS
CO_50_4	50	4	0.030
CO_50_12	50	12	0.014
CO_50_24	50	24	0.001
CO_80_4	80	4	0.091
CO_80_12	80	12	0.104
CO_80_24	80	24	0.088
CO_100_4	100	4	0.132
CO_100_12	100	12	0.210
CO_100_24	100	24	0.204

**Table 2:** The (002) peak position, maximum intensity, FWHM, crystallinity index, and crystallite size of cellulose and cellulose oleates.

Sample	(002) Peak Position (2θ)	Max Intensity (counts/s)	FWHM (2θ)	CrI (%)	Crystallite Size(nm)
MCC	22.99	141	1.92	86.30	4.41
CO_50_4	22.33	250	1.92	82.17	4.41
CO_50_12	22.83	162	1.92	82.63	4.41
CO_50_24	22.39	186	2.11	79.75	4.01
CO_80_4	22.62	223	2.11	82.97	4.01
CO_80_12	22.72	170	1.92	78.77	4.41
CO_80_24	22.79	121	1.73	84.62	4.89
CO_100_4	22.77	146	1.73	80.00	4.89
CO_100_12	22.83	157	1.92	80.83	4.41
CO_100_24	22.70	163	1.73	79.81	4.89

## Data Availability

The data presented are contained within the article.

## Supplementary Materials

The following are available online at https://www.mdpi.com/article/10.3390/polym13244397/s1, Figure S1: Solubility Test. Cellulose Oleates (COs) were dispersed by 90 min of sonication and kept static for 1 day. 20 mg of CO was dispersed in 10 mL of dimethyl sulfoxide (DMSO), *N,N*-dimethylformamide (DMF), tetrahydrofuran (THF), chloroform (CHCl_3_), diethyl ether (Et_2_O), toluene (Tol) and hexane (Hx), respectively, Figure S2: Images of comparison of microcrystalline cellulose (MCC) and COs in 10 mL of (a) DMF and (b) THF after 90 min of sonication and kept static for 1 day, Figure S3: DSC thermograms of cellulose oleates with the reaction time and temperature at 50 °C, 12 h (CO_50_12), 80 °C, 12 h (CO_80_12) and 100 °C, 12 h (CO_100_12); Table S1: Polarity index and dielectric constant of various organic solvent.
