# Supplementary material for: Esterification of Cellulose with Long Fatty Acid Chain through Mechanochemical Method
Source: Polymers (Basel). 2021 Dec 15;13(24):4397. doi: 10.3390/polym13244397 (PMC8705890; doi:10.3390/polym13244397)
Supplement: Supplementary file 1 [file polymers-13-04397-s001.zip › polymers-1486069-supplementary.pdf]

polymers

Supplementary Materials

Esterification of Cellulose with Long Fatty Acid Chain through Mechanochemical Method

Jacqueline Lease [1], Tessei Kawano [1] and Yoshito Andou [1,2,*]

[1] Department of Biological Functions Engineering, Graduate School of Life Science and Systems Engineering, Kyushu Institute of Technology, 2–4 Hibikino, Wakamatsu-ku, Kitakyushu, Fukuoka 808-0196, Japan; lease.jacqueline708@mail.kyutech.jp (J.L.); kawano.tessei758@mail.kyutech.jp (T.K.)

[2] Collaborative Research Centre for Green Materials on Environmental Technology, Kyushu Institute of Technology, 2–4 Hibikino, Wakamatsu-ku, Kitakyushu, Fukuoka 808-0196, Japan

* Correspondence: yando@life.kyutech.ac.jp

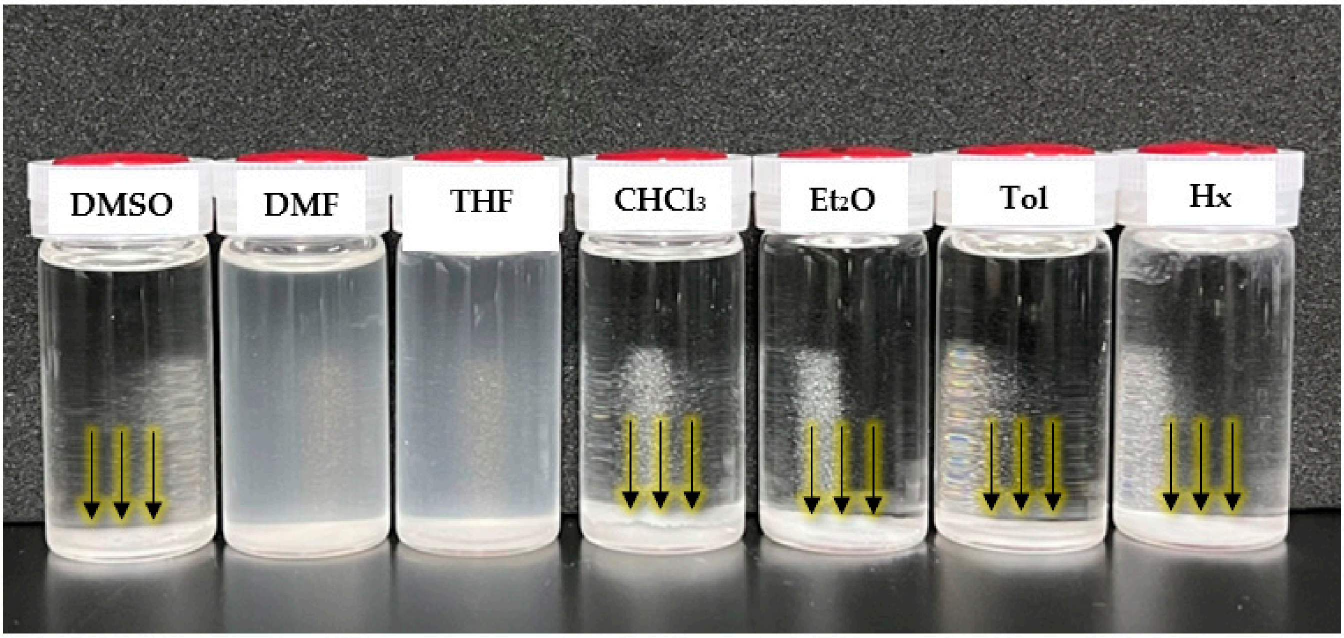

Figure S1. Solubility Test. Cellulose Oleates (COs) were dispersed by 90 minutes of sonication and kept static for 1 day. 20 mg of CO was dispersed in 10ml of dimethyl sulfoxide (DMSO), *N*,*N*-dimethylformamide (DMF), tetrahydrofuran (THF), chloroform ($CHCl_3$), diethyl ether (Et_2O), toluene (Tol) and hexane (Hx), respectively.

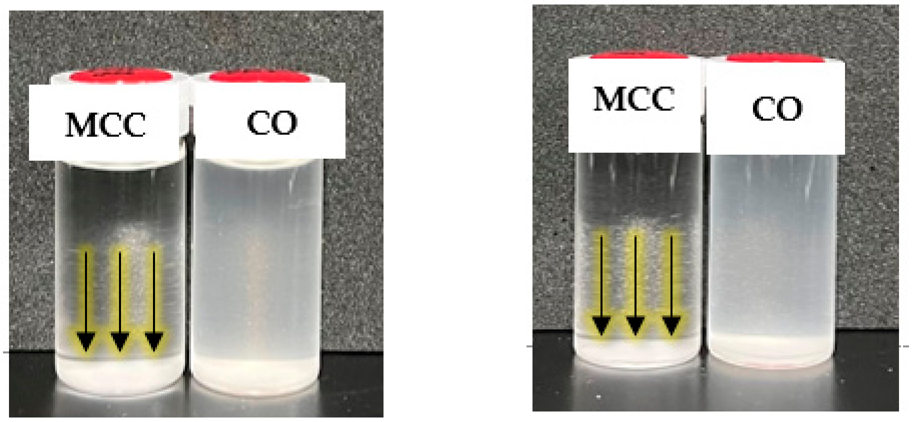

(**a**) (**b**)

Figure S2. Images of comparison of microcrystalline cellulose (MCC) and COs in 10ml of (**a**) DMF and (**b**) THF after 90 minutes of sonication and kept static for 1 day.

Table S1. Polarity index and dielectric constant of various organic solvent.

Solvent	DMSO	DMF	Hexane	Toluene	Et_2O	$CHCl_3$	THF
**Polarity Index**	7.2	6.4	0.1	2.4	2.8	4.1	4.0
**Dielectric Constant**	46.68	36.71	1.88	2.38	4.33	4.81	7.58

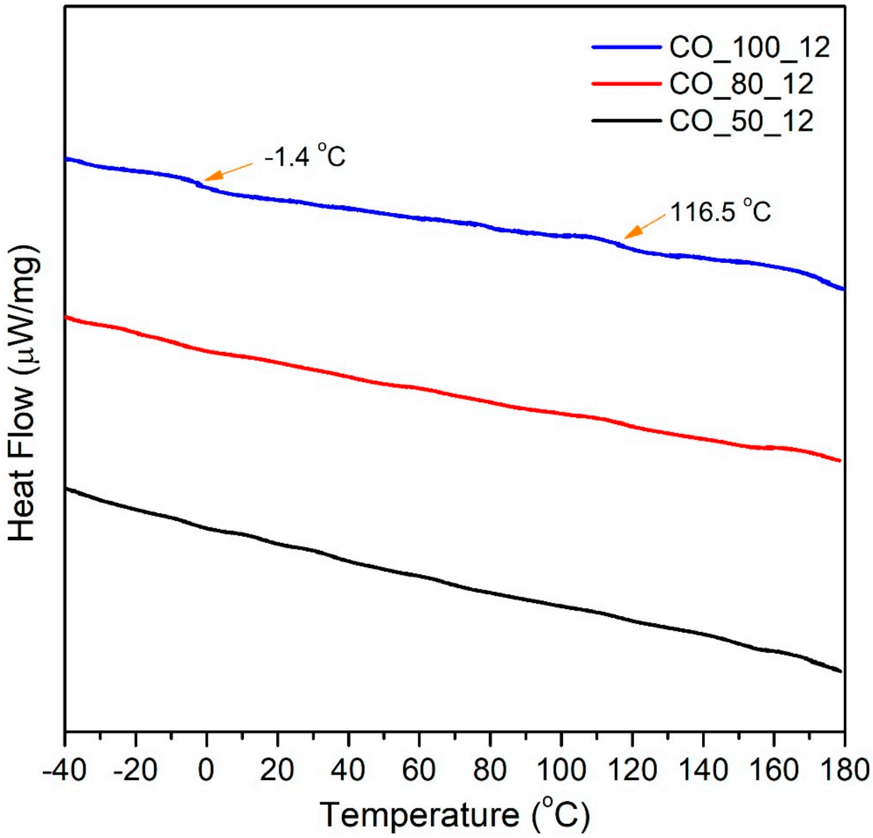

Figure S3. DSC thermograms of cellulose oleates with the reaction time and temperature at 50 °C, 12 hours (CO_50_12), 80 °C, 12 hours (CO_80_12) and 100°C, 12 hours (CO_100_12).
